# Low Trauma Fractures in People With HIV: Longitudinal Time Trends in the Swiss HIV Cohort Study, 2009–2022

**DOI:** 10.1093/cid/ciaf392

**Published:** 2025-07-17

**Authors:** Isabella C Schoepf, Vera Baltisberger, Christian W Thorball, Lene Ryom, Gilles Wandeler, David Haerry, Christian R Kahlert, Enos Bernasconi, Alexandra Calmy, Olivier Lamy, Jacques Fellay, Huldrych F Günthard, Bruno Ledergerber, Philip E Tarr, for the Swiss HIV Cohort Study, I A Abela, K Aebi-Popp, A Anagnostopoulos, M Battegay, E Bernasconi, D L Braun, H C Bucher, A Calmy, M Cavassini, A Ciuffi, G Dollenmaier, M Egger, L Elzi, J S Fehr, J Fellay, H Furrer, C A Fux, H F Günthard, A Hachfeld, D H U Haerry, B Hasse, H H Hirsch, M Hoffmann, I Hösli, M Huber, D Jackson-Perry, C R Kahlert, O Keiser, T Klimkait, R D Kouyos, H Kovari, K Kusejko, N D Labhardt, K Leuzinger, Martinez B de Tejada, C Marzolini, K J Metzner, N Müller, J Nemeth, D Nicca, J Notter, P Paioni, G Pantaleo, M Perreau, A Rauch, L P Salazar-Vizcaya, P Schmid, O Segeral, R F Speck, M Stöckle, P E Tarr, A Trkola, G Wandeler, M Weisser, S Yerly

**Affiliations:** University Center for Internal Medicine and Infectious Diseases Service, Kantonsspital Baselland, University of Basel, Bruderholz, Switzerland; Department for Visceral Surgery and Medicine, Inselspital, Bern University Hospital, University of Bern, Bern, Switzerland; University Center for Internal Medicine and Infectious Diseases Service, Kantonsspital Baselland, University of Basel, Bruderholz, Switzerland; Precision Medicine Unit, Lausanne University Hospital and University of Lausanne, Lausanne, Switzerland; School of Life Sciences, Ecole Polytechnique Fédérale de Lausanne, Lausanne, Switzerland; Center of Excellence for Health, Immunity and Infections, Rigshospitalet, University of Copenhagen, Copenhagen, Denmark; Department of Infectious Diseases, Hvidovre University Hospital, Copenhagen, Denmark; Department of Clinical Medicine, University of Copenhagen, Copenhagen, Denmark; Department of Infectious Diseases, Bern University Hospital, University of Bern, Bern, Switzerland; Positive Council, Zurich, Switzerland; Division of Infectious Diseases, Kantonsspital St Gallen, St Gallen, Switzerland; Division of Infectious Diseases, Ente Ospedaliero Cantonale, Lugano, University of Geneva, Geneva, Switzerland; Università Della Svizzera Italiana, Lugano, Switzerland; Division of Infectious Disease, Geneva University Hospital, Geneva, Switzerland; Bone Unit, Lausanne University Hospital, University of Lausanne, Lausanne, Switzerland; Precision Medicine Unit, Lausanne University Hospital and University of Lausanne, Lausanne, Switzerland; School of Life Sciences, Ecole Polytechnique Fédérale de Lausanne, Lausanne, Switzerland; Department of Infectious Diseases and Hospital Epidemiology, University Hospital Zurich, University of Zurich, Zurich, Switzerland; Institute of Medical Virology, University of Zurich, Zurich, Switzerland; Department of Infectious Diseases and Hospital Epidemiology, University Hospital Zurich, University of Zurich, Zurich, Switzerland; University Center for Internal Medicine and Infectious Diseases Service, Kantonsspital Baselland, University of Basel, Bruderholz, Switzerland

**Keywords:** HIV infection, low trauma fractures, longitudinal time trends, aging, multivariable analysis

## Abstract

**Background:**

People with human immunodeficiency virus (HIV) are at increased risk of low trauma fractures (LTFs). Published data on LTF incidence trends over time have not been uniform. This study sought to analyze LTF time trends in the Swiss HIV Cohort Study (SHCS) over the time period 2009–2022.

**Methods:**

Fractures are prospectively captured in the SHCS. Since 2008, using a standardized form, each fracture and its low trauma nature was validated by the treating HIV physician and the main investigators. Applying negative binomial regression, we estimated the LTF incidence rate ratio per calendar year univariably and adjusting for time-updated clinical and HIV-related risk factors, plus a genome-wide polygenic risk score associated with bone mineral density.

**Results:**

Between 2009 and 2022, 7524 SHCS participants accumulated 71 983 participant-years of observation and 235 validated LTFs, for an LTF incidence of 0.33 (95% confidence interval [CI], .29–.37) per 100 participant-years. There were statistically significant changes over time in multiple demographic, clinical, and HIV-related variables potentially associated with better bone health. The LTF incidence rate declined by 9.2% (95% CI, 5.6%–12.6%) per year on average in univariable analysis and by 7.5% (95% CI, 2.9%–11.9%) per year in the full multivariable model. Declining LTF time trends were noted in men and women, younger and older age groups, and participants with favorable and unfavorable genetic background.

**Conclusions:**

LTFs have considerably decreased in people with HIV in Switzerland over a 14-year period. The LTF decline likely is multifactorial and occurred concomitant with favorable trends in antiretroviral therapy, demographic, and lifestyle variables that may contribute to better bone health.

## Teaser

In the Swiss HIV Cohort Study, the adjusted low trauma fracture incidence rate declined 7.5% yearly from 2009 to 2022. The decline was similar in men and women, younger and older participants, and those with favorable and unfavorable genetic background.

## Graphical Abstract

**Graphical Abstract:**
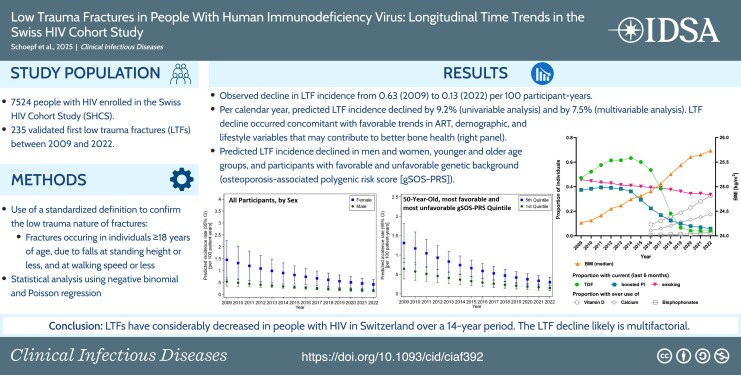
https://tidbitapp.io/institutional-portal/clinical-infectious-diseases/tidbits/low-trauma-fractures-in-people-with-hiv-longitudinal-time-trends-in-the-swiss-hiv-cohort-study/update

Bone health is a major long-term concern in people with human immunodeficiency virus (HIV). Current guidelines suggest that people with HIV have an increased risk of osteoporosis and low trauma fractures (LTFs) compared to the general population, and LTFs appear 10 years earlier in people with HIV [1–3]. Increased susceptibility to osteoporosis and LTF in people with HIV has been attributed to a higher prevalence of risk factors such as low body weight, vitamin D deficiency, hepatitis C virus (HCV) coinfection, smoking and other substance use, chronic inflammation, and toxicity from antiretroviral therapy (ART) agents including tenofovir disoproxil fumarate (TDF) and boosted protease inhibitors (bPIs). It is therefore important that management of bone health in people with HIV is implemented at an early stage, including periodic assessment of fracture risk, promotion of a physically active lifestyle, sufficient intake of vitamin D and calcium, and selection of ART in favor of bone-friendly options [1–5].

Information on LTF incidence time trends in people with HIV is limited. Published studies from the United States and Europe have shown no clear LTF incidence time trends [6], a decrease over time [7, 8], or even a recent increase [9]. Because these results have not been uniform, here we analyze time trends in LTFs and in demographic, clinical, and HIV-related variables associated with bone health in the Swiss HIV Cohort Study (SHCS) from 2009 to 2022. Fractures are prospectively recorded in the SHCS using a standardized form, and we analyzed only fractures whose low trauma nature was validated by the HIV physician and the investigators using a standardized definition [10].

## METHODS

### Study Population

The study was approved by the respective local ethics committees. Participants were enrolled in the SHCS (www.shcs.ch) [11] and gave informed consent for study participation including genetic testing. Because LTF validation was conducted as part of a genetic LTF study in the SHCS (analyses ongoing), and previous LTF genome-wide association studies were conducted in populations of mostly European descent, the study was limited to participants with self-reported European ancestry. Bone mineral density (BMD) assessments are not done systematically in SHCS participants. LTF cases included all SHCS participants who sustained an LTF during the study period (2009–2022). Only the first LTF was considered for those experiencing multiple LTFs, and participants with fractures before 2009 were excluded. SHCS participants who remained free of fractures throughout the study period were considered potential controls.

### Low Trauma Fractures

Fractures are prospectively captured in the SHCS. Since 2008, the SHCS has used a standardized form with which each fracture is adjudicated, including its low trauma nature, date, and body site [10]. Each LTF was validated by the treating HIV physician and the main investigators. LTFs were defined as reported previously [10], that is, LTFs occurred at age ≥18 years, at walking speed or less, and from a fall that occurred at standing height or less. Because fractures of the skull, cervical spine, fingers, and toes are typically considered traumatic in nature, these were excluded. To avoid including pathological fractures, fractures occurring in persons with a history of malignancy were excluded. Asymptomatic vertebral fractures detected by imaging were included, and diagnosis relied on the attending radiologist's report using as fracture date the imaging date.

### Time-Updated Clinical Characteristics

Clinical characteristics included age, sex, menopause, transmission group, median body mass index (BMI), BMI category (underweight, normal, overweight/obese) [12], diabetes mellitus, treatment with corticosteroids (ever used oral prednisone 5 mg equivalent for ≥3 months) [13], parental hip fracture history [14], alcohol consumption (>3 standard units daily consumption vs less) [15], smoking (current, past, never), physical activity, active injection drug use (IDU), and ever use of vitamin D, calcium, and bisphosphonates. HIV-related risk factors included current CD4 cell count, CD4 nadir, CD4 count at ART start, HCV seropositivity, HCV RNA detectable, years since ART start, HIV RNA suppressed, and maximal log HIV viremia. ART exposures were defined a priori, based on their LTF association in large observational studies [11, 12], and included cumulative exposure (years), never exposed, current exposure <6 months, current exposure <2 years, and current exposure ≥2 years to TDF or bPIs.

### Genotyping and Genome-wide Polygenic Risk Score

We applied a polygenic risk score (PRS), referred to as gSOS (genetically predicted heel quantitative ultrasound speed of sound), that has been associated with low BMD. The gSOS-PRS has been extensively validated in the general population [16] and was recently validated by us to be associated with BMD also in people with HIV in Switzerland [17]. For details on genotyping including quality control measures and PRS construction, please refer to Schwenke et al [17].

### Statistical Analyses

In an open cohort analysis, we investigated time trends of validated first LTF from 2009 onward, as prospective recording of LTFs started in August 2008 in the SHCS. The study period was from 2009 to 2022. Persons without LTF were included if they had at least 2 follow-up visits and no follow-up gaps of >2 years. Time at risk was calculated from the first visit after 2008 until the diagnosis of LTF or the last follow-up, whichever occurred first. Due to overdispersion, we applied negative binomial regression to estimate the LTF incidence rate ratio (IRR) per calendar year and to predict the yearly incidence in all SHCS participants, separately in male and female participants (sex at birth), separately for participants aged 40, 50, and 60 years, and separately for participants in the extreme (most favorable vs most unfavorable) quintiles of the gSOS-PRS [17].

Since we counted participants in every year at risk with time-updated clinical characteristics measured yearly on 1 July, we applied robust standard errors accounting for repeated measures. Because of the limited number of LTF events, we grouped calendar time into 3-year intervals, except the last interval, which included 2 years. Time trends of clinical and HIV-related characteristics were analyzed with linear regression using robust standard errors. We further evaluated whether the observed calendar-year time-trends are altered by including time-updated clinical and HIV-related risk factors. Data analysis was performed with Stata/SE version 18.0 software (StataCorp, College Station, Texas, USA).

### Sensitivity Analyses

We repeated these analyses applying, first, Poisson regression instead of negative binomial regression and, second, a closed cohort format constituted as a subgroup of the open cohort with participants enrolled prior to 2009 but with new participants not allowed to enter the dataset.

## RESULTS

### Participants, Fractures

Between 2009 and 2022, 7524 SHCS participants had accumulated 71 983 participant-years of observation and 235 validated LTFs. Fracture sites (multiple sites possible) included spine (n = 125), femoral neck (n = 49), lower leg (n = 42), foot (without toes, n = 33), ribs (n = 33), wrist (n = 13), pelvis (n = 2), patella (n = 1), and other sites (n = 19). Participant characteristics are shown in Table 1. Time trends for clinical variables and antiretroviral exposures for the entire SHCS population are shown in Table 2 and Supplementary Table 1. Participants with LTF were more likely to be female, older, active injection drug users, physically inactive, exposed to corticosteroids ≥3 months, currently exposed to TDF and bPIs, current smokers, HCV coinfected, to have a parental history of hip fracture, and to have lower CD4 values.

**Table 1.** Characteristics of 7524 Swiss HIV Cohort Study Participants (Open Cohort)

Characteristic	Participants		
	All Participants (n = 7524)	Participants With a First LTF (n = 235)	Participants Without LTF (n = 7289)
Male sex	6246 (83)	157 (66.8)	6089 (83.5)
Age, y, median (IQR)	55 (46–62)	56 (50–64)	55 (46–62)
Menopause^a^	803/1278 (62.8)	59/78 (75.6)	744/1200 (62)
Fracture date, median (IQR)	…	27 Aug 2014 (7 Apr 2011–19 Feb 2018)	…
Transmission group			
MSM	4230 (56.2)	82 (34.9)	4148 (56.9)
Heterosexual	2169 (28.8)	81 (34.5)	20 886 (28.6)
IDU	790 (10.5)	61 (26)	729 (10)
Other/unknown	335 (4.5)	11 (4.7)	324 (4.4)
BMI, kg/m^2^			
Underweight (<18.5)	291 (3.4)	20 (8.5)	271 (3.7)
Normal (18.5–24.9)	3610 (47.6)	125 (53.2)	3485 (47.8)
Overweight/obese (≥25)	3623 (48.8)	90 (38.3)	3533 (48.5)
Physical activity^b,c^			
<1×/wk	3800/7285 (52.2)	129/197 (65.5)	3671/7088 (51.8)
≥1×/wk	3485/7285 (47.8)	68/197 (34.5)	3417/7088 (48.2)
Diabetes mellitus	573 (7.6)	16 (6.8)	557 (7.6)
Corticosteroid use ≥3 mo	282 (3.7)	27 (11.5)	255 (3.5)
Parental hip fracture	324 (2.5)	24 (6)	300 (2.6)
BMD assessed using DEXA	2386 (33.7)	90 (37.9)	2296 (31.5)
Smoking			
Never	2387 (31.7)	61 (26)	2326 (31.9)
Current	2803 (37.3)	112 (47.7)	2691 (36.9)
Past	2334 (31)	62 (26.4)	2272 (31.2)
Maximum alcohol intake			
None/mild	3967 (52.7)	121 (51.5)	3846 (52.8)
Moderate/heavy	3557 (47.3)	114 (48.5)	3443 (47.2)
Active IDU	1049 (13.9)	74 (31.5)	975 (13.4)
HCV seropositivity	1279 (17)	86 (36.6)	1193 (16.4)
HCV RNA detectable^d^	392/6899 (5.7)	21/183 (11.5)	371/6716 (5.5)
Current CD4 count, cells/μL, median (IQR)^e^	690 (501–902)	482 (321–723)	694 (509–907)
CD4 at ART start, cells/μL, median (IQR)	275 (149–415)	207.5 (90–314)	278 (151–418)
CD4 nadir, cells/μL, median (IQR)	230 (115–348)	129 (39–218)	233 (120–352)
CD4 nadir <200 cells/μL	3226 (42.9)	161 (68.5)	3065 (42)
HIV RNA max, log median (IQR)	5.03 (4.4–5.6)	5.19 (4.7–5.8)	5.03 (4.4–5.5)
HIV RNA <50 copies/mL	7179 (95.4)	215 (91.5)	6964 (95.5)
Years since ART initiation, median (IQR)	13 (8–21)	14 (7–18)	13 (8–21)
TDF exposure			
Never	1774 (23.6)	54 (23)	1720 (23.6)
Past exposure <6 mo	281 (3.7)	6 (2.6)	275 (3.8)
Past exposure ≥6 mo	4402 (58.5)	77 (32.8)	4325 (59.3)
Current exposure <6 mo	23 (0.3)	7 (3)	16 (0.2)
Current exposure ≥6 mo	1044 (13.9)	91 (38.7)	953 (13.1)
bPI exposure			
Never	3190 (42.6)	58 (25)	3132 (43.1)
Past exposure <6 mo	433 (5.8)	7 (3)	426 (5.9)
Past exposure ≥6 mo	3056 (40.8)	62 (26.7)	2994 (41.2)
Current exposure <6 mo	6 (0.08)	1 (0.4)	5 (0.07)
Current exposure ≥6 mo	897 (10.8)	104 (44.8)	703 (9.7)

Table shows characteristics of study participants in the middle of the last year each patient contributed information. Data are presented as No. (%) unless otherwise indicated.

Abbreviations: ART, antiretroviral therapy; BMD, bone mineral density; BMI, body mass index; bPI, boosted protease inhibitor; DEXA, dual energy x-ray absorptiometry; HCV, hepatitis C virus; HIV, human immunodeficiency virus; IDU, injection drug use; IQR, interquartile range; LTF, low trauma fracture; MSM, men who have sex with men; TDF, tenofovir disoproxil fumarate.

^a^Menopause status was considered only for female participants (n = 1278).

^b^38 LTF cases and 201 controls with missing values.

^c^Captured as number of ≥20-minute activity units in past 6 months.

^d^52 LTF cases and 573 controls with missing values.

^e^27 LTF cases and 1062 controls with missing values.

**Table 2.** Descriptive Longitudinal Time Trends and Trend Analysis of Clinical Characteristics in the Entire Swiss HIV Cohort Study Population, Stratified by Calendar Year (20092022), and Grouped into 3-Year Intervals, Except for 2021–2022 (2-Year Interval)

Characteristic	Open Cohort					Time Trend Analysis Annual Change (in percent, unless indicated otherwise)	*P* Value
	2009–2011	2012–2014	2015–2017	2018–2020	2021–2022	2009–2022	
Participants, No.	13 641	15 154	16 374	16 957	11 033	…	
LTFs, No.	71	51	52	43	18	…	
Age, y, median (IQR)	47 (41–53)	49 (43–55)	51 (44–57)	53 (45–59)	55 (46–61)	.53 y	<.01
Female sex	2721 (20)	2815 (18.6)	2865 (17.5)	2814 (16.6)	1738 (15.8)	−.3	<.01
Menopause^a^	725 (26.6)	1107 (39.3)	1421 (49.6)	1664 (59.1)	1139 (65.5)	.004	<.01
Transmission group							
MSM	7001 (51.3)	8275 (54.6)	9261 (56.6)	9900 (58.4)	6590 (59.7)	…	
Heterosexual	4255 (31.2)	4535 (29.9)	4788 (29.2)	4873 (28.7)	3096 (28.1)	…	
IDU	1891 (13.9)	1780 (11.8)	1655 (10.1)	1451 (8.6)	853 (7.7)	…	
Other/unknown	494 (3.6)	564 (3.7)	670 (4.1)	733 (4.3)	494 (4.5)	…	
BMI, kg/m^2^							
Underweight (<18.5)	582 (4.3)	570 (3.8)	601 (3.7)	498 (2.9)	292 (2.7)	…	
Normal (18.5–24.9)	7995 (58.6)	8519 (56.2)	8644 (52.8)	8221 (48.5)	5111 (46.3)	…	
Overweight/obese (≥25)	5064 (37.1)	6065 (40)	7129 (43.5)	8238 (48.6)	5630 (51)	…	
Median (IQR)	23.8 (21.7–26.4)	24.1 (21.9–26.8)	24.4 (22.1–27.1)	24.9 (22.5–27.8)	25.1 (22.7–28)	.1 (kg/m^2^)	<.01
Physical activity^b^							
<1×/wk	4884/8685 (56.2)^c^	8091/15 024 (53.9)^d^	7963/16 259 (49)^e^	7869/16 815 (46.8)^f^	5474/10 958 (50)^g^	−0.7	<.01
≥1×/wk	3801/8685 (43.8)^c^	6933/15 024 (46.1)^d^	8296/16 259 (51)^e^	8946/16 815 (53.2)^f^	5484/10 958 (50)^g^	0.7	<.01
Diabetes mellitus	673 (4.9)	784 (5.2)	900 (5.5)	1054 (6.2)	775 (7)	.2	<.01
Corticosteroid use ≥3 mo	590 (4.3)	622 (4.1)	629 (3.8)	622 (3.7)	379 (3.4)	−.1	<.01
Parental hip fracture	661 (4.9)	707 (4.7)	754 (4.6)	810 (4.8)	528 (4.8)	0	.94
Smoking							
Never	4235 (31.1)	4882 (32.2)	5382 (32.9)	5666 (33.4)	5717 (33.7)	…	
Current	6077 (44.6)	6316 (41.7)	6467 (39.5)	6108 (36)	3746 (34)	−.9	<.01
Past	3329 (24.4)	3956 (26.1)	4525 (27.6)	5183 (30.6)	3570 (32.4)	…	
Maximum alcohol intake							
None/mild	12 700/13 627 (93.2)^h^	12 949/15 154 (85.5)^i^	13 707/16 368 (83.7)^j^	14 179/16 950 (83.7)^k^	9434/11 029 (85.5)^l^	…	
Moderate/heavy	927/13 627 (6.8)^h^	2192/15 154 (14.5)^i^	2661/16 368 (16.3)^j^	2771/16 950 (16.3)^k^	1595/11 029 (14.5)^l^	.2	<.01
Active IDU	2334 (17.1)	2283 (15.1)	2181 (13.3)	2007 (11.8)	1200 (10.9)	−.5	<.01
HCV seropositivity	2607 (19.1)	2602 (17.2)	2583 (15.8)	2496 (14.7)	1557 (14.1)	−.4	<.01
HCV RNA detectable	762 (6.3)^m^	804 (5.9)^n^	841 (5.6)^o^	795 (5.1)^p^	453 (4.6)^q^	<.01	<.01
Current CD4 count, cells/μL, median (IQR)	552 (408–733)	607 (454–799)	659 (496–853)	707 (534–912)	714 (542–924)	15 (cells/μL)	<.01
CD4 at ART start, cells/μL, median (IQR)	249 (130–361)^r^	258 (140–378)^s^	269 (150–401)^t^	280 (159–425)^u^	285 (160–434)^v^	…	
CD4 nadir, cells/μL, median (IQR)	198 (91–291)	209 (102–306)	223 (115–329)	236 (124–354)	243 (129–367)	5 (cells/μL)	<.01
CD4 nadir <200 cells/μL	6880 (50.4)	7157 (47.2)	7172 (43.8)	6944 (41)	4352 (39.5)	−1	<.01
HIV RNA max, log median (IQR)	5.12 (4.58–5.59)	5.1 (4.57–5.57)	5.06 (4.5–5.55)	5.03 (4.44–5.53)	5.01 (4.4–5.53)	−2	<.01
HIV RNA <50 copies/mL	11 391 (83.5)	13 908 (91.8)	15 660 (95.6)	16 437 (96.9)	10 713 (97.1)	1.1	<.01
Years since ART initiation, median (IQR)	9 (3–13)	9 (4–16)	10 (5–18)	12 (7–20)	14 (8–22)	.6 y	<.01
Use of vitamin D, ever^w^	17 (0.12)	350 (2.3)	1526 (9.3)	3240 (19.1)	3338 (30.3)	3.9	<.01
Use of calcium, ever^w^	20 (0.15)	146 (1)	739 (4.5)	1841 (10.9)	1811 (16.4)	2.3	<.01
Use of bisphosphonate ever^w^	2 (0.01)	14 (0.1)	90 (0.6)	227 (1.3)	269 (2.4)	.4	<.01

**Figure 1.**
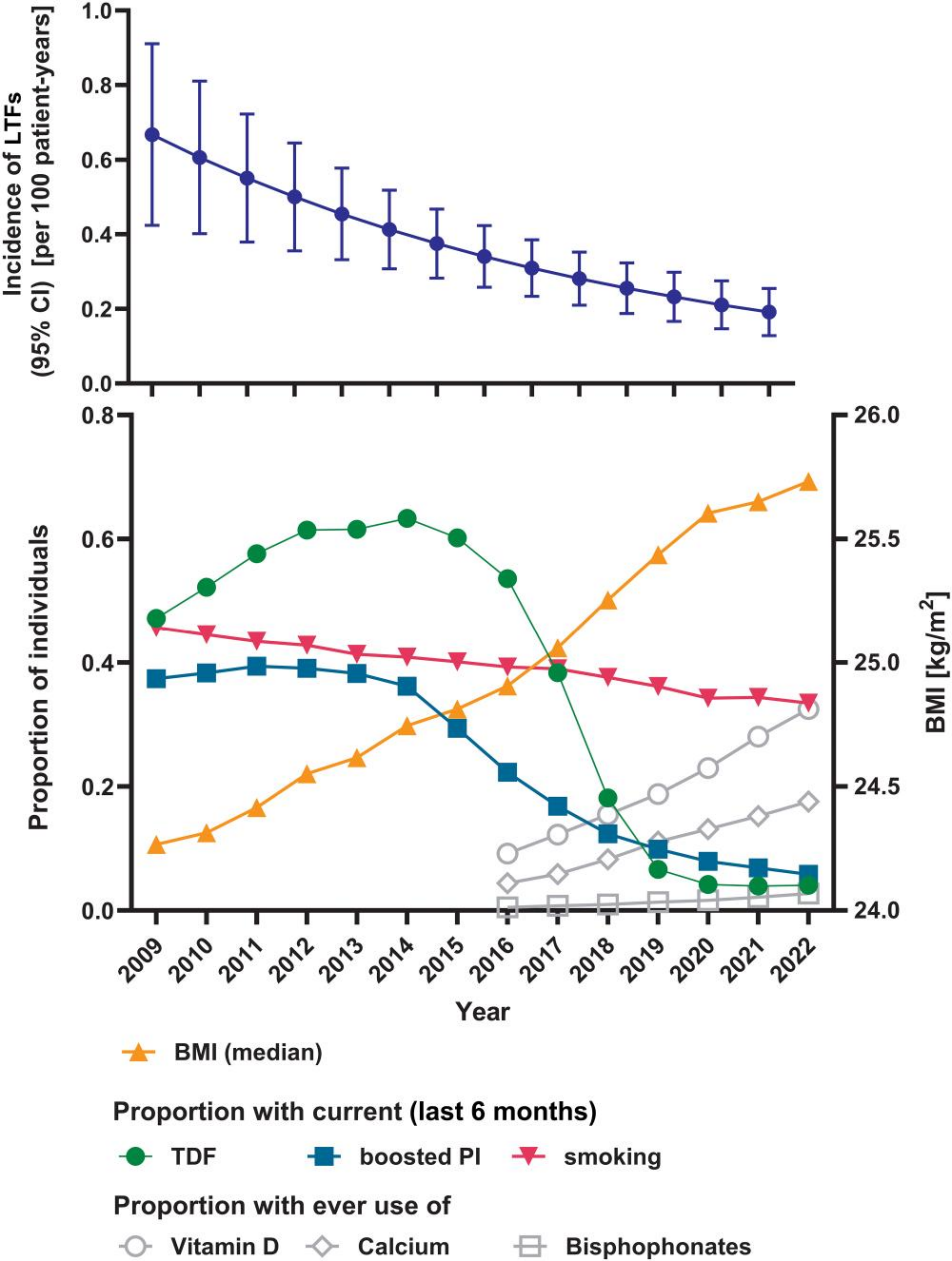
Predicted low trauma fracture (LTF) incidence rate per year between 2009 and 2022 in 7524 Swiss HIV Cohort Study (SHCS) participants, (open cohort), including 71 983 participant-years of observation and 235 validated LTFs. Predicted LTF incidence rate ratio declined by 9.2% per year. Clinical characteristics shown represent figures for the entire SHCS population and are shown at the middle of each calendar year. From 2009 to 2022, median body mass index, and ever use of vitamin D, calcium, and bisphosphonate (for participants with LTF: ever used prior to date of LTF) increased. From 2009 to 2022, current smoking and current exposure to tenofovir disoproxil fumarate and boosted protease inhibitors decreased. The data visualized in Figure 1 are also tabulated in Table 2 and Supplementary Table 1. Time trends regarding different clinical characteristics in the entire SHCS population are shown in Table 2 and Supplementary Table 1, and results for the closed cohort analysis are shown in Supplementary Tables 2 and 3. Abbreviations: BMI, body mass index; CI, confidence interval; LTF, low trauma fracture; PI, protease inhibitor; TDF, tenofovir disoproxil fumarate.

Data are presented as No. (%) unless otherwise indicated; the time trend analysis annual change is shown as percentage unless otherwise indicated. Time trends regarding the main clinical characteristics from Table 1 are displayed in Figure 1.

Abbreviations: ART, antiretroviral therapy; BMI, body mass index; HCV, hepatitis C virus; HIV, human immunodeficiency virus; IDU, injection drug use; IQR, interquartile range; LTF, low trauma fracture; MSM, men who have sex with men.

^a^Menopause status was considered only for female participants.

^b^Captured as number of ≥20-minute activity units in past 6 months.

^c^4956 missing values.

^d^130 missing values.

^e^115 missing values.

^f^142 missing values.

^g^75 missing values.

^h^14 missing values.

^i^13 missing values.

^j^6 missing values.

^k^7 missing values.

^l^4 missing values.

^m^1563 missing values.

^n^1505 missing values.

^o^1442 missing values.

^p^1295 missing values.

^q^751 missing values.

^r^1418 missing values.

^s^1619 missing values.

^t^1882 missing values.

^u^2158 missing values.

^v^1515 missing values.

^w^For LTF cases: prior to date of LTF.

### LTF Incidence 2009–2022: Observed Data

The LTF incidence during the study period was 0.33 (95% confidence interval [CI], .29–.37) per 100 participant-years. The LTF incidence was 0.63 (95% CI, .41–.91) in 2009, and declined to 0.13 (95% CI, .05–.27) in 2022.

### Predicted LTF Incidence Time Trends (2009–2022)

In univariable analysis, the predicted LTF incidence rate declined by 9.2% per calendar year (LTF IRR per calendar year, 0.908 [95% CI, .874–.944]; *P* < .01; Figure 1). This LTF incidence time trend was not significantly altered when we added the time-updated clinical variables (Table 2) and ART exposures (Supplementary Table 1) in a multivariable model. The adjusted LTF incidence rate declined by 7.5% per calendar year (LTF IRR per calendar year, 0.925 [95% CI, .881–.971]; *P* < .01; Table 3).

**Table 3.** Estimated Low Trauma Fracture Incidence Rate Ratio (2009–2022) per Calendar Year According to Risk Factors: Multivariable Negative Binomial Regression Analysis

Risk Factor	IRR (95% CI)	*P* Value
Per calendar year	0.925 (.881–.970)	<.01
TDF exposure		
Current exposure ≥6 mo	Reference	
Never	1.193 (.812–1.753)	.37
Past exposure <6 mo	0.779 (.302–2.011)	.61
Past exposure ≥6 mo	1.024 (.686–1.528)	.09
Current exposure <6 mo	3.284 (1.350–7.989)	<.01
bPI exposure		
Current exposure ≥6 mo	Reference	
Never	0.517 (.349–.765)	<.01
Past exposure <6 mo	0.424 (.184–.976)	.04
Past exposure ≥6 mo	0.512 (.351–.748)	<.01
Current exposure <6 mo	0.292 (.051–1.657)	.17
Current age (per year older)	1.069 (1.052–1.087)	<.01
Female sex	1.332 (.764–2.320)	.31
Menopause^a^	2.054 (1.028–4.101)	.04
Body mass index	0.995 (.959–1.033)	.8
Injection drug use	1.636 (1.012–2.646)	.04
CD4 count (per 100 cells/µL higher)	0.903 (.839–.971)	<.01
Lowest CD4 before event (per 100 cells/µL higher)	0.858 (.694–1.062)	.16
CD4 nadir <200 cells/µL	0.892 (.526–1.515)	.67
Current HIV-1 RNA <50 copies/mL	1.196 (.436–3.277)	.73
Current HIV RNA level (per log increase)	1.139 (.847–1.533)	.39
Maximum HIV RNA level (per log increase)	1.146 (.982–1.337)	.08
HCV antibody seropositivity	1.712 (1.104–2.655)	.02
Diabetes mellitus	0.972 (.518–1.824)	.93
Parental history of hip fracture	1.832 (1.080–3.107)	.03
Current smoking	1.585 (1.135–2.215)	<.01
Corticosteroid use ≥3 mo	2.614 (1.468–4.655)	<.01
Moderate to heavy alcohol intake	1.079 (.805–1.445)	.61
Hypercholesterolemia	0.828 (.611–1.123)	.22

Abbreviations: bPI, boosted protease inhibitor; CI, confidence interval; HCV, hepatitis C virus; HIV, human immunodeficiency virus; IRR, incidence rate ratio; TDF, tenofovir disoproxil fumarate.

^a^Menopause status was considered only for female participants.

### Predicted LTF Incidence Time Trends by Sex, Age, and gSOS-PRS

**Figure 2.**
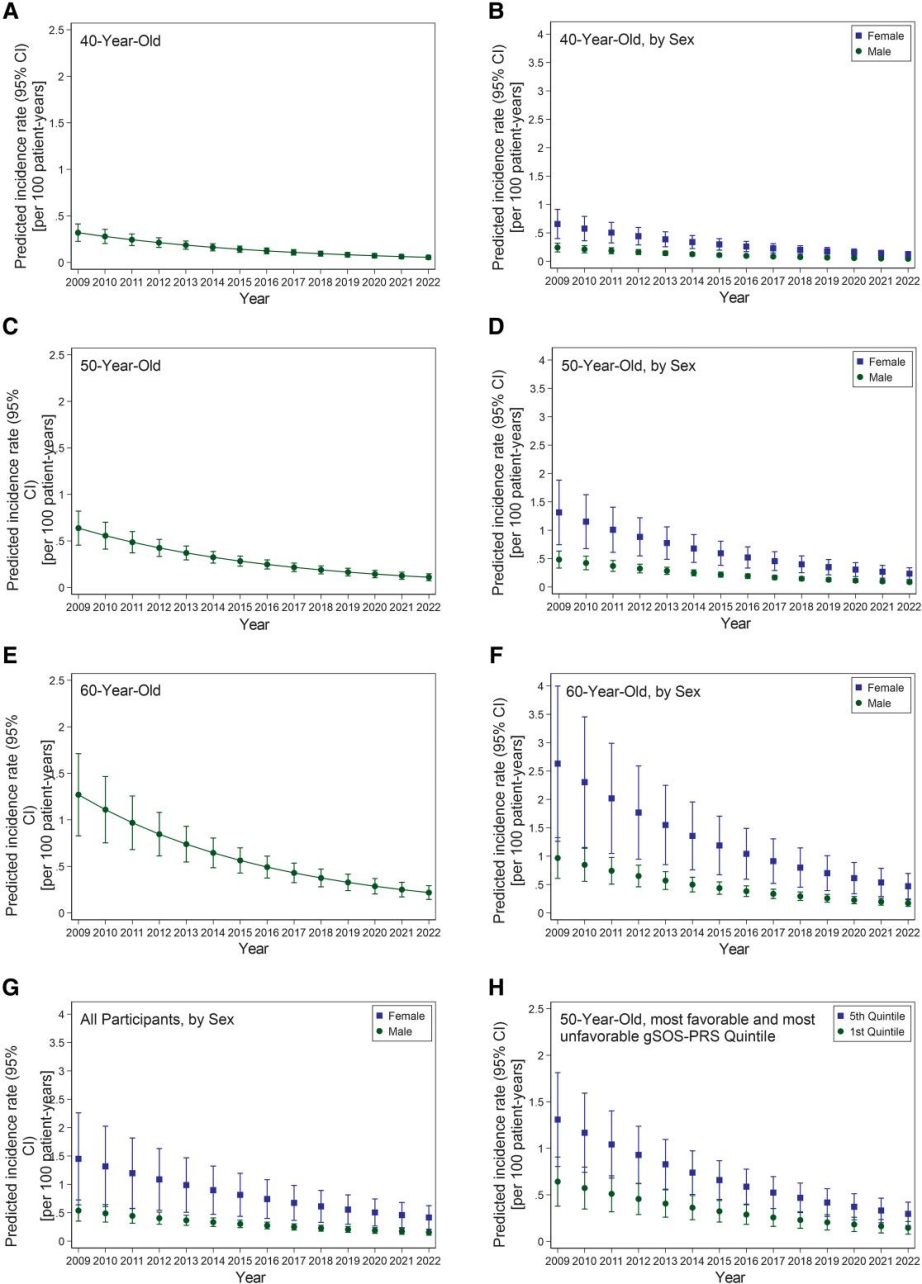
Predicted low trauma fracture (LTF) incidence rates between 2009 and 2022 in the entire Swiss HIV Cohort Study population (open cohort). *A*, Predicted LTF incidence rate for a 40-year-old person. *B*, Predicted LTF incidence rate for a 40-year-old person, shown separately for females (sex at birth) (blue squares) and males (green dots). *C*, Predicted LTF incidence rate for a 50-year-old person. *D*, Predicted LTF incidence rate for a 50-year-old person, shown separately for females (sex at birth) (blue squares) and males (green dots). *E*, Predicted LTF incidence rate for a 60-year-old person. *F*, Predicted LTF incidence rate for a 60-year-old person, shown separately for females (sex at birth) (blue squares) and males (green dots). *G*, Predicted LTF time trends, all participants, shown separately for females (sex at birth) (blue squares) and males (green dots). *H*, Predicted LTF incidence rate for a 50-year-old person in the first (most favorable) (green dots) and fifth (most unfavorable) (blue squares) genetically predicted heel quantitative ultrasound speed of sound polygenic risk score (gSOS-PRS) quintile. gSOS-PRS data were available for 4056 of 7524 participants (235/235 LTF cases and 3821/7289 LTF-free controls). There is no indication of different yearly decline by gSOS quintile (interaction analysis *P* > .2). Abbreviations: CI, confidence interval; gSOS-PRS, genetically predicted heel quantitative ultrasound speed of sound polygenic risk score.

The predicted LTF incidence rate during the study period was increased 2.69-fold (95% CI, 1.78- to 4.06-fold; *P* < .01) in females (sex at birth) compared to males (Figure 2*G*). The downward trend in LTF incidence was apparent in both younger and older participants (Figure 2*A*, 2*C*, and 2*E*), in both female and male participants (Figure 2*B*, 2*D*, 2*F*, and 2*G*), and in both participants with favorable and unfavorable genetic background regarding bone health (top and bottom quintile of the gSOS polygenic risk score; Figure 2*H*).

### Descriptive Longitudinal Trends of Clinical and ART Characteristics that may be associated with Bone and Overall Health (2009–2022, Entire SHCS)

Statistically significant increasing trends were seen regarding median age, proportion of male sex, postmenopausal women, men who have sex with men, BMI, percentage of overweight/obese participants, physically active participants, prevalence of diabetes mellitus, participants with moderate/heavy alcohol intake, current CD4 cell count, CD4 nadir, CD4 at ART start, years since ART initiation, and participants with suppressed HIV RNA (Table 2). Statistically significant decreasing trends were seen for smoking, active IDU, corticosteroid use, HCV seropositivity, maximum HIV RNA levels, detectable HCV RNA, and participants with CD4 nadir <200 cells/μL. Cumulative years of exposure to TDF or bPIs increased over time until 2021, while current TDF and bPI exposure decreased over time (Supplementary Table 1). Finally, the proportion of participants treated with supplementary vitamin D, calcium, and bisphosphonate therapy increased (Figure 1).

### Sensitivity Analyses

Time trends remained essentially unchanged when we applied a closed cohort format (n = 4994 participants; Supplementary Results, Supplementary Tables 2–4) and when we applied Poisson regression (Supplementary Tables 5 and 6).

## DISCUSSION

We report here on a 9.2% and 7.5%, respectively, univariable and multivariable adjusted annual decrease in LTF incidence in people with HIV in Switzerland over a 14-year period. From 2009 to 2022, decreasing LTF time trends were recorded in men and women, in younger and older participants, and in those with favorable and unfavorable genetic background regarding BMD. These findings provide clinically important, reassuring information regarding bone health for people with HIV and clinicians alike. Interestingly, the LTF decline occurred despite an SHCS population that is aging, and which over time has included more postmenopausal women.

We were unable to identify a unifying explanation for these decreasing LTF time trends when we adjusted for multiple time-updated clinical and HIV-related factors that have significantly changed over time. This suggests that the recent LTF decline in the SHCS is most likely multifactorial, and that each of the variables analyzed has a limited effect size on LTF incidence over time. For example, in parallel to the decreasing LTF incidence, we document significant longitudinal demographic changes (eg, fewer injection drug users, fewer underweight participants), as well as medication changes that may have contributed to better bone health. Specifically, the decreasing LTF time trends occurred in the context of increasingly well-controlled HIV infection, lifestyle improvements (less smoking, more physical activity), and de-prescribing of potentially bone-toxic medication including TDF [4, 5] but also bPIs, as recommended per international HIV guidelines [1, 2], and corticosteroids. While cumulative TDF exposure increased during 2009–2022, current and recent exposure substantially decreased, in the setting of both de-prescribing and fewer new prescriptions. Even though data on non-ART treatments is recorded in the SHCS only since 2015, we note clear increasing trends in prescriptions aimed at bone health (eg, more vitamin D, calcium, and bisphosphonates).

Our results appear robust because we included all 235 SHCS participants with LTFs who were recorded over a 14-year time period and >7000 comparison participants without LTF, with adequate data quality regarding duration of observation and missing data. Additional strengths include utilization of a standardized LTF definition during the entire study period, prospective collection of LTFs using standardized forms, and physician validation of the low trauma nature of each included fracture. Moreover, rich clinical, sociodemographic, and behavioral information was collected per protocol in a semiannual fashion in the setting of the well-established, nationally representative SHCS [11]. Importantly, results were comparable in sensitivity analyses, without any relevant dilution effect from new participants being added to the open cohort over time, and we find no evidence of any major attrition bias.

In the general population, increasing rates of osteoporosis and LTF have been recorded worldwide in the past 3 decades, which have been attributed mainly to aging populations [18]. In contrast, in the Swiss general population there was a slight decrease in LTFs from 2010 to 2019 [19]. Previous studies done in people with HIV were typically conducted during years when recommended ART regimens included bPIs and TDF and have not documented any significant temporal fracture time trends in the United States (n = 245 fractures included 1996–2008, without detail provided on the traumatic or LTF nature of the included fractures) [6]. LTF rates initially increased and then decreased in people with HIV from Denmark (n = 375 LTFs included 1996–2009 and n = 457 LTFs included 1995–2014, respectively) [7, 8]. In contrast, in people with HIV in Spain (n = 115 LTFs included), LTF incidence decreased during 2004–2016 but increased during 2016–2020 [9]. While comparison of the LTF incidence with the general population in Switzerland should be done with caution, the 0.33/100 person-years and 0.22/100 person-years LTF incidence in the SHCS (2009–2022 and 2019 alone, respectively) seems comparable to the 0.24/100 person-years incidence recorded in the Swiss general population (2019) [19].

A meta-analysis summarizing the information gathered from 26 randomized ART trials [20] reported an increased LTF rate among ART-naive participants and within the first 2 years after ART initiation (0.53/100 person-years), compared to subsequent years after ART start (0.30/100 person-years). In addition, significant bone demineralization has been observed in the first 2 years of ART, but stable BMD was noted during long-term ART [21]. This is in line with our findings where fracture rates were increased early after TDF and bPI exposure, but decreased over time despite increasing years of cumulative ART exposure.

Our study has limitations. Our analyses are limited to active SHCS participants (approximately 72% of all ART-treated people in Switzerland) [11]. Additional limitations include no information on falls; data on non-ART medication being available only since 2015, including information on hormone replacement therapy; and insufficient details (eg, diagnosis date, anti-inflammatory treatment history) regarding chronic inflammatory conditions such as rheumatoid arthritis. Our population was predominantly white, male, and middle-aged; therefore, results should be cautiously extrapolated to other populations. Even though the SHCS is a relatively young cohort (median age of 47 and 55 years in 2009–2011 and in 2021–2022, respectively; Table 2), we cannot exclude competing risks such as death [22]; that is, older participants may die before developing fractures. Finally, BMD is not measured systematically in the SHCS [17, 23]. Early detection of osteoporosis might potentially have led to earlier start of osteoporosis management and an even earlier and steeper decline in LTF incidence than we detected in this study.

In conclusion, in people with HIV in Switzerland, time trend analysis over 14 years shows a reassuring and large LTF incidence decline in both male and female participants. The decline most likely is multifactorial, and it occurred in the setting of a number of favorable trends that include ART, demographic, and lifestyle changes that may have contributed to improved bone health.

## Supplementary Material

ciaf392_Supplementary_Data

## Supplementary Data

Supplementary materials are available at *Clinical Infectious Diseases* online. Consisting of data provided by the authors to benefit the reader, the posted materials are not copyedited and are the sole responsibility of the authors, so questions or comments should be addressed to the corresponding author.

## Notes

***Author Contributions.*** Study design: I. C. S., B. L., and P. E. T. Data management and participant selection: B. L. Data acquisition: I. C. S., G. W., C. R. K., E. B., A. C., H. F. G., and P. E. T. Data analysis: I. C. S., V. B., B. L., and P. E. T. Drafting of the manuscript: I. C. S., V. B., and P. E. T. Critical review and revision of the manuscript: All authors.

***Swiss HIV Cohort Study members.*** Abela IA, Aebi-Popp K, Anagnostopoulos A, Battegay M, Bernasconi E, Braun DL, Bucher HC, Calmy A, Cavassini M (Chairman of the Clinical and Laboratory Committee), Ciuffi A, Dollenmaier G, Egger M, Elzi L, Fehr JS, Fellay J, Furrer H, Fux CA, Günthard HF, Hachfeld A, Haerry DHU (Deputy of Positive Council), Hasse B, Hirsch HH, Hoffmann M, Hösli I, Huber M, Jackson-Perry D (patient representatives), Kahlert CR (Chairman of the Mother and Child Substudy), Keiser O, Klimkait T, Kouyos RD, Kovari H, Kusejko K (Head of Data Center), Labhardt ND, Leuzinger K, Martinez de Tejada B, Marzolini C, Metzner KJ, Müller N, Nemeth J, Nicca D, Notter J, Paioni P, Pantaleo G, Perreau M, Rauch A (President of the SHCS), Salazar-Vizcaya LP, Schmid P, Segeral O, Speck RF, Stöckle M, Tarr PE, Trkola A, Wandeler G (Chairman of the Scientific Board), Weisser M, and Yerly S.

***Acknowledgments.*** The authors acknowledge the effort and commitment of the investigators, study nurses, laboratory personnel, and participants.

***Financial support.*** This work has been financed within the framework of the Swiss HIV Cohort Study (SHCS), supported by the Swiss National Science Foundation (grant number 33FI-0_229621) and by the SHCS Research Foundation. The data are gathered by the 5 Swiss university hospitals, 2 cantonal hospitals, 15 affiliated hospitals, and 36 private physicians (listed in https://www.shcs.ch/health-care-providers/).
