# Supplementary material for: Low Trauma Fractures in People With HIV: Longitudinal Time Trends in the Swiss HIV Cohort Study, 2009–2022
Source: Clin Infect Dis. 2025 Jul 17;81(6):e581–90. doi: 10.1093/cid/ciaf392 (PMC13471031; doi:10.1093/cid/ciaf392)
Supplement: Supplementary file 1 [file ciaf392_supplementary_data.docx]

**SUPPLEMENTARY DATA**

**Schoepf IC et al. Low Trauma Fractures in People with HIV: Longitudinal Time Trends in the Swiss HIV Cohort Study**

**Supplementary Results**

**LTF Participants (Closed Cohort).** Between 2009 and 2022, 5005 SHCS participants had accumulated 56’278 participant-years of observation and 212 validated LTFs (LTF incidence, 0.38 [95% CI, 0.33–0.43] per 100 participant-years).

**Predicted LTF Incidence Time Trends in the Closed Cohort.** Predicted LTF incidence rate declined by 7% (95% CI, 3.2-10.7%; p<0.01) per year in univariable analysis, and by 11.7% (95% CI, 8.1-15.2%) per year adjusting for age. Predicted LTF incidence rate was increased 2.59-fold (95% CI, 1.68–3.99; p<0.01) in females (sex at birth) compared to males.

**Descriptive Longitudinal Trends of Clinical Characteristics (2009–2022) in the Closed Cohort.** Trends of clinical characteristics stratified by calendar year are shown in **Supplementary Table 2** and **3**. From 2009 to 2022, median age, male sex, postmenopausal women, MSM, BMI, and the percentage of overweight/obese participants *increased*, as did physical activity (except in 2021/2022), diabetes mellitus, current CD4 cells, CD4 at nadir, CD4 at ART start, years since ART initiation, and participants with suppressed HIV RNA. In addition, there was an increase in alcohol consumption (except in 2021/2022), intake of corticosteroids, supplementary Vitamin D, calcium, and of bisphosphonate therapy. From 2009 to 2022, *decreasing* trends were seen for smoking, intravenous drug use, maximal HIV RNA levels, HCV RNA detectable, and participants with CD4 nadir <200cells/ul. Cumulative years of exposure to tenofovir disoproxil fumarate (TDF), or boosted protease inhibitors (BPI) increased over time, while current exposure decreased over time. Finally, the proportion of participants treated with supplementary Vitamin D, calcium, and bisphosphonate therapy increased.

**Supplementary Table 1: Descriptive Longitudinal Time Trends and Longitudinal Trends Analysis of Tenofovir Disoproxil Fumarate (TDF) and Boosted Protease Inhibitor (BPI) Use in the entire Swiss HIV Cohort Study (SHCS) Population (open cohort) stratified by calendar years (2009–2022) grouped into 3-year intervals, except 2021-2022 (2-year interval).**

|  | **Open Cohort** | | | | | **Time Trend Analysis**  Annual change, in percent unless indicated otherwise | **P- Value** |
| --- | --- | --- | --- | --- | --- | --- | --- |
|  | **2009-2011** | **2012-2014** | **2015-2017** | **2018-2020** | **2021-2022** | **2009-2022** |  |
| TDF cumulative exposure, median years (IQR) | 1.48  (0-3.95) | 3.28  (.55-6.04) | 4.77  (1.28-7.96) | 5.04  (.51-8.76) | 4.71  (.07-8.75) | 2.7 years | <.01 |
| TDF never exposed, n (%) | 4387  (32.2) | 3105  (20.5) | 2792  (17.1) | 3572  (21.1) | 2644  (24) | -0.6 | <.01 |
| TDF current <2years, n (%) | 1812  (13.3) | 1446  (9.5) | 686  (4.2) | 54  (0.3) | 43  (0.4) | -1.3 | <.01 |
| TDF current <6 month, n (%) | 633  (4.6) | 408  (2.7) | 168  (1.0) | 11  (0.06) | 14  (0.13) | -0.4 | <.01 |
| TDF current >2years, n (%) | 5347  (39.2) | 7971  (52.6) | 7600  (46.4) | 1584  (9.3) | 402  (3.6) | -3.9 | <.01 |
| BPI cumulative exposure, median years (IQR) | 1.08  (0-4.35) | 1.53  (0-5.75) | 1.41  (0-6.65) | 0.76  (0-6.7) | 0.47  (0-6.38) | 0.1 years | <.01 |
| BPI never exposed, n (%) | 5013  (36.8) | 5227  (34.5) | 6030  (36.8) | 7078  (41.7) | 4886  (44.3) | .07 | <.01 |
| BPI current <2years, n (%) | 885  (6.5) | 695  (4.6) | 173  (1.1) | 22  (0.1) | 11  (0.1) | -0.6 | <0.01 |
| BPI current <6 month, n (%) | 303  (2.2) | 216  (1.4) | 44  (0.27) | 9  (0.05) | 3  (0.03) | -0.2 | <.01 |
| BPI current >2years, n (%) | 4356  (31.9) | 5037  (33.2) | 3557  (21.7) | 1686  (9.9) | 691  (6.3) | -2.6 | <.01 |

**Supplementary Table 2: Descriptive Longitudinal Time Trends and Longitudinal Trends Analysis of Clinical Characteristics**

**in the Closed Cohort (n=4994 participants) stratified by Calendar Years (2009–2022) grouped into 3-year intervals, except 2021-2022 (2-year interval).**

|  |  | **Closed Cohort**  **Median (Interquartile Range) or No. (%)** | | | | | **Time Trend Analysis**  Annual change, in percent unless indicated otherwise | **P-Value** |
| --- | --- | --- | --- | --- | --- | --- | --- | --- |
|  |  | **2009-2011** | **2009-2022** | **2015-2017** | **2018-2020** | **2021-2022** | **2009-2022** |  |
| Participants |  | 13641 | 13380 | 12264 | 11144 | 6783 | -- | -- |
| Low Trauma Fractures, n |  | 71 | 46 | 47 | 34 | 14 | -- | -- |
| Age (years) |  | 47  (41–53) | 49  (43–56) | 52  (47–59) | 55  (50–61) | 57  (52–63) | .87 years | <.01 |
| Female sex, n (%) |  | 2721  (20) | 2587  (19.3) | 2361  (19.3) | 2141  (19.2) | 1272  (18.) | -.1 | <.01 |
| Menopause,^a^ n(%)^l^ |  | 725  (26.6) | 1107  (39.3) | 1421  (49.6) | 1664  (59.1) | 1139  (65.5) | .01 | <.01 |
| Transmission group, n (%) | Men who have Sex  with Men | 7001  (51.3) | 7106  (53.1) | 6584  (53.7) | 6078  (54.5) | 3763  (55.5) | -- | -- |
|  | Heterosexual | 4255  (31.2) | 4127  (30.8) | 3767  (30.7) | 3413  (30.6) | 2045  (30.2) | -- | -- |
|  | Injection drug use | 1891  (13.9) | 1682  (12.6) | 1477  (12) | 1259  (11.3) | 739  (10.9) | -- | -- |
|  | Other/unknown | 494  (3.6) | 465  (3.5) | 436  (3.6) | 394  (3.5) | 236  (3.5) | -- | -- |
| BMI, n (%) | Underweight  (BMI <18.5 kg/m^2^) | 582  (4.3) | 514  (3.8) | 451  (3.7) | 356  (3.2) | 208  (3.1) | -- | -- |
|  | Normal  (BMI 18.5-24.9 kg/ m^2^) | 7995  (58.6) | 7531  (56.3) | 6543  (53.4) | 5434  (48.8) | 3163  (46.6) | -- | -- |
|  | Overweight/Obese  (BMI ≥25 kg/m^2^) | 5064  (37.1) | 5335  (39.9) | 5270  (43) | 5354  (48.8) | 3412  (50.3) | -- | -- |
| BMI (median), kg/m^2^ |  | 23.8  (21.7-26.4) | 24.1  (21.9-26.7) | 24.3  (22.1-27.1) | 24.8  (22.4-27.7) | 25  (22.6-27.9) | .1 (kg/m^2^) | <.01 |
| Physical activity^b^ | <1x/week | 4884/8685 (56.2)^c^ | 7265/13265 (54.8)^d^ | 6153/12178 (50.5)^e^ | 5314/11054 (48.1)^f^ | 3516/6738  (52.2)^g^ | -.6 | <.01 |
|  | ≥1x/week | 3801/8685 (43.8)^c^ | 6000/13265 (45.2)^d^ | 6025/12178 (49.5)^e^ | 5740/11054 (51.9)^f^ | 3222/6738 (47.5)^g^ | .6 | <.01 |
| Diabetes mellitus |  | 673  (4.9) | 741  (5.5) | 766  (6.3) | 840  (7.5) | 599  (8.8) | .3 | <.01 |
| Corticosteroids ≥3 months |  | 590  (4.3) | 565  (4.2) | 508  (4.1) | 458  (4.1) | 275  (4.1) | -.02 | <.01 |
| Parent hip fracture |  | 661  (4.9) | 646  (4.8) | 608  (5) | 579  (5.2) | 360  (5.3) | .04 | <.01 |
| Smoking | never | 4235  (31.1) | 4214  (31.5) | 3858  (31.5) | 3521  (31.6) | 2129  (31.4) | -- | -- |
|  | current | 6077  (44.6) | 5533  (41.4) | 4755  (38.8) | 3907  (35.1) | 2190  (32.3) | -1 | <.01 |
|  | past | 3329  (24.4) | 3633  (27.2) | 3651  (29.8) | 3716  (33.4) | 2464  (36.3) | -- | -- |
| Maximum alcohol intake | none / mild | 4884/8685 (56.2)^h^ | 7265/13265 (54.8)^i^ | 6153/12178 (50.5)^j^ | 5314/11054 (48.1)^k^ | 3516/6738 (52.2) | -- | -- |
|  | moderate / heavy | 3801/8685 (43.8)^h^ | 6000/13265 (45.2)^i^ | 6025/12178 (49.5)^j^ | 5740/11054 (51.9)^k^ | 3222/6738 (47.5) | .3 | <.01 |
| Active injection drug use |  | 2334  (17.1) | 2124  (15.9) | 1859  (15.2) | 1587  (14.2) | 935  (13.8) | -.3 | <.01 |
| Hepatitis C Seropositivity |  | 2607  (19.1) | 2459  (18.4) | 2280  (18.6) | 2076  (18.6) | 1279  (18.9) | -.02 | .56 |
| HCV RNA detectable,  n (%) |  | 762  (6.3)^l^ | 733  (6.1)^m^ | 699  (6.3)^n^ | 620  (6.1)^o^ | 365  (5.9)^p^ | <.01 | .41 |
| Current CD4 (cells/μL), median |  | 552  (408-733) | 610  (460-802) | 662  (500-855) | 702  (534-902) | 705  (539-911) | 14 (cells/μL) | <.01 |
| CD4 (cells/μL) at ART start, median (IQR) |  | 249  (130-361)^q^ | 251  (134-365)^r^ | 253  (137-366)^s^ | 255  (140-369)^t^ | 257  (142.5-371)^u^ | -- | -- |
| CD4 nadir (cells/μL), median |  | 198  (91-291) | 200  (94-290) | 200  (97-288) | 201  (100-288) | 204  (100-290) | .2 (cells/μL) | .1 |
| CD4 nadir <200 cells/μl |  | 6880  (50.4) | 6632  (49.6) | 6076  (49.5) | 5483  (49.2) | 3294  (48.6) | .1 | <.01 |
| HIV RNA max, log median |  | 5.12  (4.58-5.59) | 5.12  (4.6-5.59) | 5.12  (4.62-5.59) | 5.13  (4.62-5.59) | 5.13  (4.61-5.59) | .3 | <.01 |
| HIV RNA<50 copies/mL |  | 11391  (83.5) | 12417  (92.8) | 11796  (96.2) | 10833  (97.2) | 6586  (97.1) | 1.2 | <.01 |
| Years since antiretroviral therapy initiation, median (IQR) years |  | 9  (3-13) | 11  (5-16) | 14  (8-19) | 17  (11-22) | 19  (14-24) | .9 years | <.01 |
| Use of Vitamin D, ever^v^ n (%) |  | 17  (0.12) | 307  (2.3) | 1215  (9.9) | 2327  (20.9) | 2194  (32.4) | 4.1 | <.01 |
| Use of Calcium, ever^v^ n (%) |  | 20  (0.15) | 136  (1) | 654  (5.3) | 1488  (13.4) | 1369  (20.2) | 2.8 | <.01 |
| Use of Bisphosphonat ever^v^ n (%) |  | 2  (0.01) | 14  (0.1) | 76  (0.6) | 191  (1.7) | 220  (3.2) | .5 | <.01 |

^a^Menopause status was considered only for female participants. ^b^captured as number of ≥20 min activity units in past 6 months. ^c^4956 missing values; ^d^115 missing values; ^e^86 missing values; ^f^90 missing values; ^g^45 missing values; ^h^14 missing values; ^i^5 missing values; ^j^3 missing values; ^k^3 missing values; ^l^1’563 missing values; ^m^1'370 missing values; ^n^1'175 missing values; ^o^986 missing values; ^p^583 missing values; ^q^1’418 missing values ^r^1’337 missing values; ^s^1’170 missing values; ^t^1’060 missing values; ^u^655 missing values; ^v^ for LTF cases: prior to date of LTF.

**Supplementary Table 3: Descriptive Longitudinal Time Trends and Longitudinal Trends Analysis of Tenofovir Disoproxil Fumarate (TDF) and Boosted Protease Inhibitor (BPI) Use in the Closed Cohort (n=4994 participants) stratified by Calendar Years (2009–2022) grouped into 3-year intervals, except 2021-2022 (2-years interval).**

|  | **Closed Cohort** | | | | | **Time Trend Analysis**  Annual change, in percent unless indicated otherwise | **P-Value** |
| --- | --- | --- | --- | --- | --- | --- | --- |
|  | **2009-2011** | **2012-2014** | **2015-2017** | **2018-2020** | **2021-2022** | **2009-2022** |  |
| TDF exposure, median years (IQR) | 1.5  (0-4) | 3.8  (.6-6.4) | 6  (2.1-8.8) | 7.1  (2.9-10) | 7.2  (3-10.2) | .43 years | <.01 |
| TDF never exposed, n (%) | 4387  (32.2) | 2770  (20.7) | 2016  (16.4) | 1742  (15.6) | 1045  (15.4) | -1.5 | <.01 |
| TDF current <2years, n (%) | 1812  (13.3) | 775  (5.8) | 231  (1.9) | 13  (0.1) | 7  (0.1) | -1.2 | <.01 |
| TDF current <6 month, n (%) | 633  (4.6) | 171  (1.3) | 51  (0.4) | 3  (0.03) | 3  (0.04) | -0.4 | <.01 |
| TDF current >2years, n (%) | 5347  (39.2) | 7425  (55.5) | 5932  (48.4) | 1149  (10.3) | 251  (3.7) | -3.5 | <.01 |
| BPI exposure, median years (IQR) | 1.1  (0-4.4) | 2.1  (0-6.3) | 2.9  (0-8.3) | 3  (0-9.1) | 2.9  (0-9.4) | 0.25 years | <.01 |
| BPI never exposed, n (%) | 5013  (36.8) | 4437  (33.2) | 3925  (32) | 3571  (32) | 2193  (32.3) | -0.4 | <.01 |
| BPI current <2years, n (%) | 885  (6.5) | 317  (2.4) | 41  (0.3) | 2  (0.1) | 2  (0.1) | -0.5 | <.01 |
| BPI current <6 month, n (%) | 303  (2.2) | 77  (0.58) | 10  (0.08) | 0  (0) | 1  (0.01) | -0.2 | <.01 |
| BPI current >2years, n (%) | 4356  (31.9) | 4800  (35.9) | 3030  (24.7) | 1420  (12.7) | 549  (8.1) | -2.3 | <.01 |

**Supplementary Table 4: Estimated Low Trauma Fracture (LTF) Incidence Rate Ratio (IRR) per Calendar year according to Risk Factors, Multivariable Negative binomial Regression Analysis (Closed Cohort, n=4994 participants)**

| **Risk Factor** |  | **IRR (95% CI)** | **p-Value** |
| --- | --- | --- | --- |
| Calendar Year |  | 0.923 (0.875 – 0.974) | <.01 |
| Tenofovir disoproxil fumarate (TDF) exposure | Current exposure ≥6 months | Reference |  |
|  | Never | 1.130 (0.750 – 1.703) | .56 |
|  | Past exposure <6 months | 0.949 (0.346 – 2.603) | .92 |
|  | Past exposure ≥6 months | 1.044 (0.682 – 1.597) | .84 |
|  | Current exposure <6 months | 2.671 (1.024 – 6.968) | .05 |
| Boosted PI exposure | Current exposure ≥6 months | Reference |  |
|  | Never | 0.488 (0.319 – 0.745) | <.01 |
|  | Past exposure <6 months | 0.431 (0.175 – 1.061) | .07 |
|  | Past exposure ≥6 months | 0.494 (0.332 – 0.735) | <.01 |
|  | Current exposure <6 months | 0.313 (0.059 – 1.670) | .17 |
| Current Age |  | 1.068 (1.050 – 1.087) | <.01 |
| Female sex |  | 1.222 (0.680 – 2.196) | .5 |
| Menopause^a^ |  | 2.361 (1.129 – 4.934) | .02 |
| Body Mass Index (BMI) |  | 1.005 (0.967 – 1.045) | .8 |
| Intravenous drug use (IVD) |  | 1.660 (1.016 – 2.714) | .04 |
| CD4 count (per 100 cells/µL higher) |  | 0.905 (0.837 – 0.978) | .01 |
| Lowest CD 4 before event (per100 cells/µL higher) |  | 0.846 (0.669 – 1.070) | .16 |
| CD4 nadir < 200 cells/µL |  | 0.894 (0.515 – 1.554) | .69 |
| Undetectable HIV-RNA (current HIV-1 RNA < 50 copies/mL) |  | 1.038 (0.364 – 2.956) | .95 |
| Current HIV-RNA level (per log increase) |  | 1.140 (0.832 – 1.562) | .41 |
| Maximum HIV-RNA level (per log increase) |  | 1.088 (0.931 – 1.273) | .29 |
| Hepatitis-C-Virus-Antibody Seropositivity |  | 1.746 (1.108 – 2.749) | .02 |
| Diabetes mellitus (Type II) |  | 0.827 (0.420 – 1.630) | .58 |
| Parental history of hip fracture |  | 1.899 (1.067 – 3.381) | .03 |
| Current smoking |  | 1.558 (1.100 – 2.207) | .01 |
| Corticosteroids >3 months |  | 2.584 (1.435 – 4.654) | <.01 |
| Maximum alcohol intake |  | 0.993 (0.728 – 1.353) | .96 |
| Hypercholesterinemia |  | 0.776 (0.560 –1.074) | .13 |

^a^Menopause status was considered only for female participants.

Note: Estimates are transformed only in the first equation to incidence-rate ratios.

CI Confidence Interval; IRR Incidence Rate Ratio; PI Protease inhibitor; RNA Ribonucleic Acid

**Supplementary Table 5: Estimated Low Trauma Fracture (LTF) Incidence Rate Ratio (IRR) per Calendar year (2009-2022) according to Risk Factors, Multivariable Poisson Regression Analysis (Open cohort, n=7524 participants)**

| **Risk Factor** |  | **IRR (95% CI)** | **p-Value** |
| --- | --- | --- | --- |
| Calendar Year |  | 0.931 (0.890 – 0.973) | <.01 |
| Tenofovir disoproxil fumarate (TDF) exposure | Current exposure ≥6 months | Reference |  |
|  | Never | 1.211 (0.856 – 1.714) | .28 |
|  | Past exposure <6 months | 0.802 (0.347 – 1.856) | .61 |
|  | Past exposure ≥6 months | 1.034 (0.720 – 1.485) | .86 |
|  | Current <6 Months | 3.543 (1.629 – 7.707) | <.01 |
| Boosted PI exposure | Current exposure ≥6 months | Reference |  |
|  | Never | 0.544 (0.380 – 0.779) | <.01 |
|  | Past exposure <6 months | 0.438 (0.203 – 0.946) | .04 |
|  | Past exposure ≥6 months | 0.538 (0.381 – 0.758) | <.01 |
|  | Current exposure <6 months | 0.385 (0.055 – 2.674) | .33 |
| Current Age |  | 1.065 (1.050 – 1.080) | <.01 |
| Female sex |  | 1.353 (0.807 – 2.268) | .25 |
| Menopause^a^ |  | 1.727 (0.976 – 3.057) | .06 |
| Body Mass Index (BMI) |  | 0.997 (0.962 – 1.033) | .89 |
| Intravenous drug use (IVD) |  | 1.562 (1.011 – 2.414) | .04 |
| CD4 count (per 100 cells/µL higher) |  | 0.906 (0.847 – 0.969) | <.01 |
| Lowest CD 4 before event (per100 cells/µL higher) |  | 0.863 (0.708 – 1.053) | .15 |
| CD4 nadir < 200 cells/µL |  | 0.866 (0.527 – 1.422) | .57 |
| Undetectable HIV-RNA (current HIV-1 RNA < 50 copies/mL) |  | 1.136 (0.480 – 2.687) | .77 |
| Current HIV-RNA level (per log increase) |  | 1.141 (0.881 – 1.477) | .32 |
| Maximum HIV-RNA level (per log increase) |  | 1.130 (0.981 – 1.301) | .09 |
| Hepatitis-C-Virus-Antibody Seropositivity |  | 1.695 (1.127 – 2.550) | .01 |
| Diabetes mellitus (Type II) |  | 0.940 (0.540 – 1.639) | .83 |
| Parental history of hip fracture |  | 1.644 (1.063 – 2.541) | .03 |
| Current smoking |  | 1.492 (1.105 – 2.014) | <.01 |
| Corticosteroids >3 months |  | 2.275 (1.491 – 3.473) | <.01 |
| Maximum alcohol intake |  | 1.084 (0.832 – 1.412) | .55 |
| Hypercholesterinemia |  | 0.849 (0.647 – 1.115) | .24 |

^a^Menopause status was considered only for female participants.

CI Confidence Interval; IRR Incidence Rate Ratio; PI Protease inhibitor; RNA Ribonucleic Acid

**Supplementary Table 6: Estimated Low Trauma Fracture (LTF) Incidence Rate Ratio (IRR) per Calendar year (2009-2022) according to Risk Factors, Multivariable Poisson Regression Analysis (Closed Cohort, n=4994 participants)**

| **Risk Factor** |  | **IRR (95% CI)** | **p-Value** |
| --- | --- | --- | --- |
| Calendar Year |  | 0.931 (0.886 – 0.977) | <.01 |
| Tenofovir disoproxil fumarate (TDF) exposure | Current exposure ≥6 months | Reference |  |
|  | Never | 1.163 (0.807 – 1.675) | .42 |
|  | Past exposure <6 months | 0.944 (0.405 – 2.202) | .9 |
|  | Past exposure ≥6 months | 1.045 (0.713 – 1.530) | .82 |
|  | Current exposure <6 months | 3.152 (1.373 – 7.236) | <.01 |
| Boosted PI exposure | Current exposure ≥6 months | Reference |  |
|  | Never | 0.518 (0.353 – 0.761) | <.01 |
|  | Past exposure <6 months | 0.447 (0.196 – 1.020) | .06 |
|  | Past exposure ≥6 months | 0.521 (0.363 – 0.748) | <.01 |
|  | Current exposure <6 months | 0.429 (0.062 – 2.945) | .39 |
| Current Age |  | 1.063 (1.047 – 1.079) | <.01 |
| Female sex |  | 1.251 (0.727 – 2.153) | .42 |
| Menopause^a^ |  | 1.927 (1.056 – 3.517) | .03 |
| Body Mass Index (BMI) |  | 1.006 (0.969 – 1.045) | .75 |
| Intravenous drug use (IVD) |  | 1.578 (1.010 – 2.465) | .05 |
| CD4 count (per 100 cells/µL higher) |  | 0.907 (0.845 – 0.973) | <.01 |
| Lowest CD 4 before event (per100 cells/µL higher) |  | 0.856 (0.690 – 1.062) | .16 |
| CD4 nadir < 200 cells/µL |  | 0.864 (0.515 – 1.448) | .58 |
| Undetectable HIV-RNA (current HIV-1 RNA < 50 copies/mL) |  | 1.021 (0.423 – 2.464) | .96 |
| Current HIV-RNA level (per log increase) |  | 1.147 (0.877 – 1.500) | .32 |
| Maximum HIV-RNA level (per log increase) |  | 1.080 (0.935 – 1.247) | .3 |
| Hepatitis-C-Virus-Antibody Seropositivity |  | 1.717 (1.125 – 2.620) | .01 |
| Diabetes mellitus (Type II) |  | 0.822 (0.450 – 1.502) | .52 |
| Parental history of hip fracture |  | 1.671 (1.058 – 2.640) | .03 |
| Current smoking |  | 1.458 (1.067 – 2.990) | .02 |
| Corticosteroids >3 months |  | 2.243 (1.437 – 3.502) | <.01 |
| Maximum alcohol intake |  | 1.007 (0.764 – 1.329) | .96 |
| Hypercholesterinemia |  | 0.805 (0.605 – 1.069) | .13 |

^a^Menopause status was considered only for female participants.

CI Confidence Interval; IRR Incidence Rate Ratio; PI Protease inhibitor; RNA Ribonucleic Acid
